# Bizarre depressed skull fracture by a tile fragment in a young child, causing superior sagittal sinus injury

**DOI:** 10.4103/2152-7806.69379

**Published:** 2010-09-16

**Authors:** Jacob Eapen Mathew, Alok Sharma

**Affiliations:** Department of Neurosurgery, Christian Medical College, Ludhiana – 141 008, Punjab, India; 1 LTMG Hospital and LTMG Medical College, Sion (West), Mumbai – 400 022, Maharashtra, India

**Keywords:** Depressed skull fracture, tile fragment, superior sagittal sinus

## Abstract

**Background:**

Head injuries following fall from height are not very uncommon in developing countries due to a lack of safety standards. We describe this bizarre injury by a tile fragment penetrating the superior sagittal sinus (SSS) and its successful surgical management.

**Case Description:**

A 7-year-old child presented with a tile fragment embedded in the skull, penetrating SSS. Urgent exploration and removal of the foreign body was done to prevent complications like infection and delayed development of intracranial hypertension. Although bleeding from the SSS was a problem, this was tackled by raising the head end and giving pressure with Surgicel and Gelatine sponge. This ensured a favorable outcome.

**Conclusion:**

Although compound depressed fractures of the SSS are managed conservatively due to the risk of fatal venous hemorrhage, the unique nature of the injury in this case warranted surgical management. This case illustrates that even in such a scenario, adherence to neurosurgical principles can ensure a good outcome.

## CASE REPORT

Head injuries following fall from height is not very uncommon in developing countries due to a lack of safety standards. A variety of foreign bodies penetrating the cranium has been described in the literature and it often requires operative intervention. However the management of compound depressed fractures of the superior sagittal sinus (SSS) is generally non operative in view of the inherent surgical risks. We describe this bizarre injury by a tile fragment causing a depressed skull fracture over the SSS and its successful surgical management.

### Case Details

**Figure 1:**
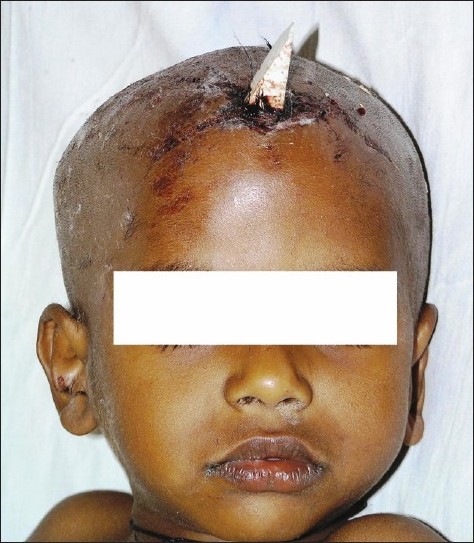
Tile fragment embedded in the skull

**Figure 2:**
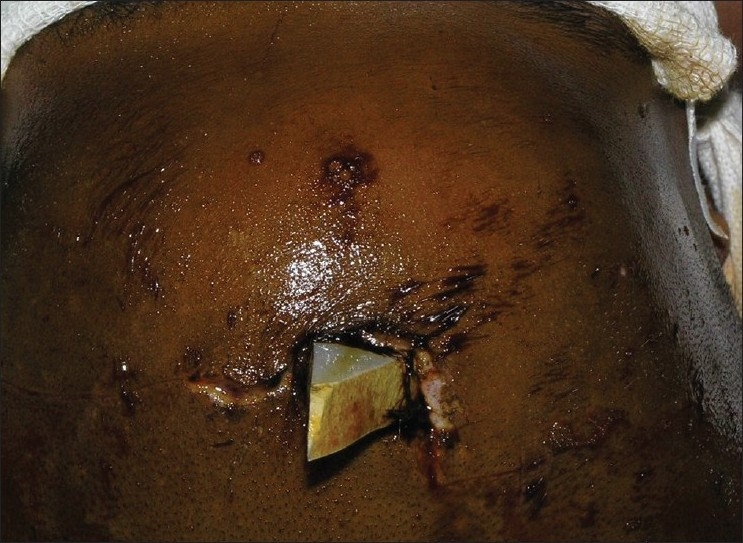
View from the top of the head

A 7-year-old child presented with a history of fall from a height of 10 feet from the roof of his house, 2 hours prior to presentation. He had a history of transient loss of consciousness. On examination, vitals signs were stable, Glasgow Coma Score (GCS) score was 15/15 and pupils were equal and reacting. There was no evidence of systemic injury. Local examination showed that a piece of tile fragment had penetrated the skull in the midline just anterior to the coronal suture [Figures 1 and 2].

**Figure 3:**
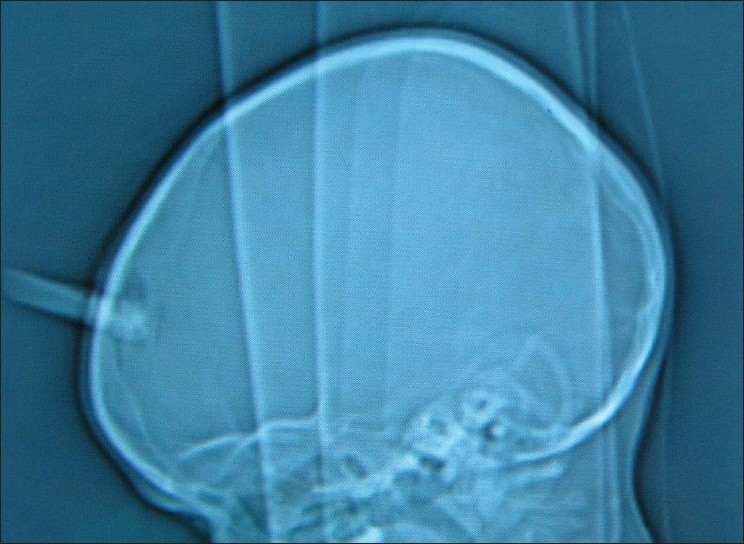
Skull X-ray showing the extent of penetration of the fragment into the skull

**Figure 4:**
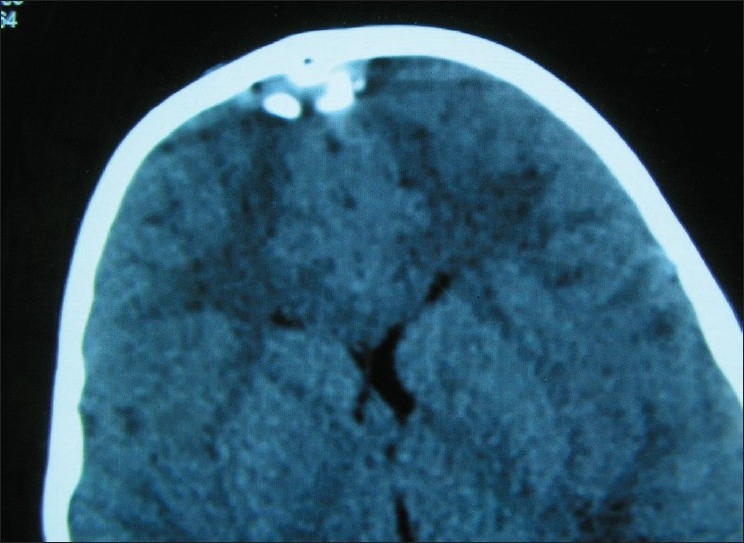
CT brain showing the compression of the SSS by the tile fragment

**Figure 5:**
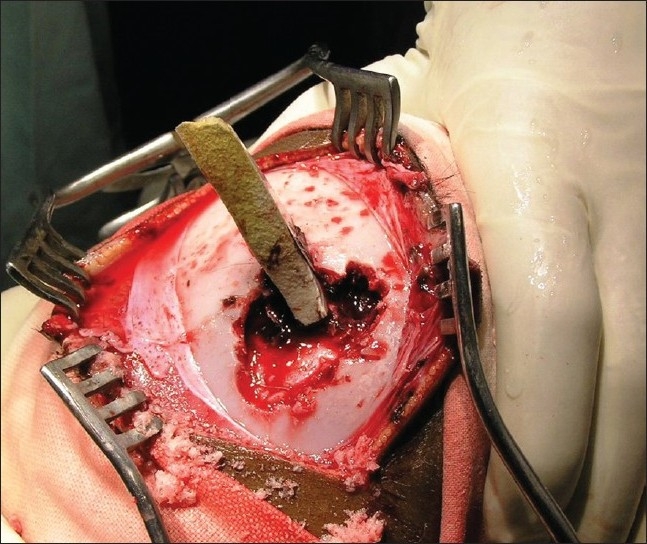
Intraoperative view showing the epidural hematoma around the FB

Lateral skull X-rays and computed tomography (CT) of the head revealed that the foreign body (FB) had penetrated 1.5 cm deep to the inner table through a fracture of the skull in the midline [Figures 3 and 4]. We suspected penetration of the SSS. He was taken up for urgent exploration and removal of the FB. Head end was raised about 30° and continuous saline irrigation was done while a burr hole and craniectomy was done around the FB. There was extradural clot around the FB [Figure 5]. On carefully removing the FB, there was bleeding from the SSS which was controlled with Gelfoam, Surgicel and pressure. After washing the wound, it was closed in layers over a drain. No anticoagulant therapy was used in the peri-operative period. Postoperative period was uneventful and he had no neurological deficits, evidence of infection or symptoms of raised intracranial pressure (suggestive of SSS thrombosis) at the time of discharge. The family was advised to bring the child for cranioplasty at a later date. However, he was lost to follow-up.

## DISCUSSION

Depressed skull fractures involving the SSS are generally treated conservatively as attempts at surgical elevation can cause fatal venous hemorrhage.[7] However, not all cases can be managed conservatively as some cases involve FBs like nails[8] or stones.[1] Some depressed fractures can cause SSS thrombosis[9–11] leading to late deterioration.

Surgery might also be warranted in rare instances of delayed intracranial hypertension in such depressed fractures overlying the SSS, as reported by Fuentes *et al*.[6] and Donovan.[5] Our patient warranted early surgical treatment in view of contaminated FB to prevent infection and delayed development of intracranial hypertension. Surgical treatment was successful in this unusual case owing to adherence to basic neurosurgical principles of managing SSS injuries, namely, raising the head end, copious irrigation of saline to prevent air embolism and patient pressure tamponade with Surgicel and Gelfoam.

A few additional points merit mention here. The sagittal sinus can be safely transected in its anterior one third, should this become necessary. The sagittal sinus can be repaired in its middle and posterior one third. A bone island surrounding the FB can be created with the help of a high speed drill and the SSS can be mobilized distally and proximally. This can be followed by a direct repair of the SSS. It is also possible to resect the SSS and replace it with a vein or dural graft although it is technically very demanding.[2–4]
